# Sucrose Utilization for Improved Crop Yields: A Review Article

**DOI:** 10.3390/ijms22094704

**Published:** 2021-04-29

**Authors:** Oluwaseun Olayemi Aluko, Chuanzong Li, Qian Wang, Haobao Liu

**Affiliations:** 1 Tobacco Research Institute of Chinese Academy of Agricultural Sciences, Qingdao 266101, China; aluko.oluseun@gmail.com (O.O.A.); lcz911006@163.com (C.L.); 2 Graduate School of Chinese Academy of Agricultural Sciences, Beijing 100081, China

**Keywords:** photosynthetic carbon assimilation, source-to-sink relationship, sucrose transporters, sucrose utilization, environmental factors, sucrose transports

## Abstract

Photosynthetic carbon converted to sucrose is vital for plant growth. Sucrose acts as a signaling molecule and a primary energy source that coordinates the source and sink development. Alteration in source–sink balance halts the physiological and developmental processes of plants, since plant growth is mostly triggered when the primary assimilates in the source leaf balance with the metabolic needs of the heterotrophic sinks. To measure up with the sink organ’s metabolic needs, the improvement of photosynthetic carbon to synthesis sucrose, its remobilization, and utilization at the sink level becomes imperative. However, environmental cues that influence sucrose balance within these plant organs, limiting positive yield prospects, have also been a rising issue over the past few decades. Thus, this review discusses strategies to improve photosynthetic carbon assimilation, the pathways actively involved in the transport of sucrose from source to sink organs, and their utilization at the sink organ. We further emphasize the impact of various environmental cues on sucrose transport and utilization, and the strategic yield improvement approaches under such conditions.

## 1. Introduction

Plant growth and development are adversely affected when photo-assimilates are not appropriately apportioned. A balanced distribution and allocation of carbon (C) to various plant organs is crucial for plant growth, since the translocation of carbohydrates from the photosynthesizing “source” leaves provide substrates required for the growth of nonphotosynthesizing “sink” organs. During photosynthesis, carbon dioxide (CO_2_) can be efficiently converted into 3-phosphoglyceric acid and glyceraldehyde-3-phosphate, which act as a precursor for starch and sucrose biosynthesis. A portion of the plant photosynthates is stored in the form of starch within the chloroplast. At night, these stored reserves are remobilized as sucrose to support plant growth and metabolism [1,2]. Sucrose is the transportable form of carbon predominantly utilized at the sink to supply the energy required for plant biomass production [3].

**Figure 1:**
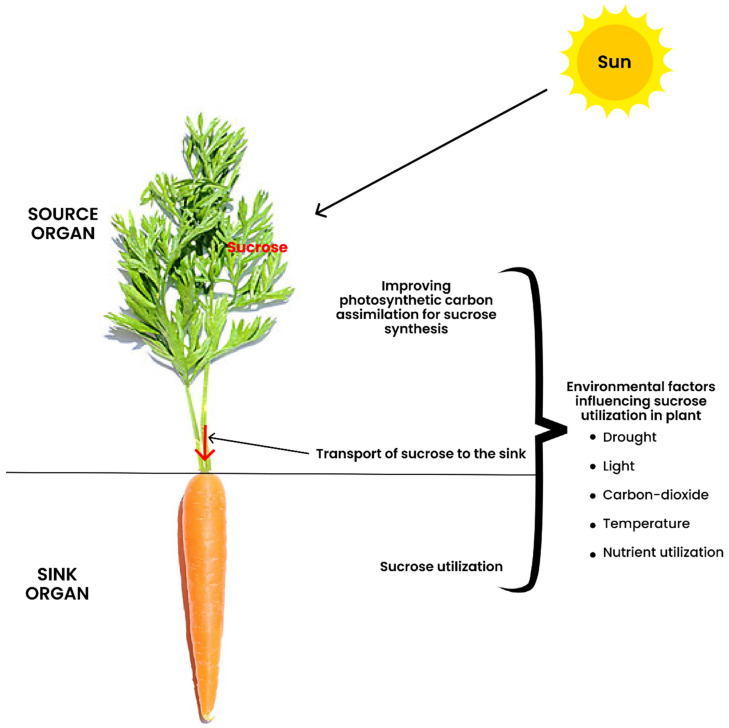
Sucrose utilization at the source and sink level. To fully optimize crop yields through sucrose utilization, improving photosynthetic carbon assimilation for sucrose synthesis, transport of sucrose to the sink, and its utilization at the sink level become imperative. Given the efficient utilization of sucrose, other environmental factors can disrupt sucrose distribution within plant organs.

Sucrose also acts as the signaling molecule that coordinates roots (sink) and shoots (mainly source) development [4,5]. Alteration in the sucrose source–sink balance impedes plant growth and development [6]. Plant growth is greatly enhanced when the primary assimilates in the source tissues balance with the metabolic needs of the heterotrophic sinks. The translocation of photo-assimilate to the sink and its utilization at the sink level is mainly altered by environmental factors. However, little attention has been directed toward the influence of these environmental factors on photo-assimilate transport and utilization at the sink level. Thus, a conceptual understanding of the source–sink interaction is paramount for optimizing plant growth under fluctuating environmental conditions. To fully understand the interaction between the partitioning of photo-assimilates, plant growth, and environment (Figure 1), the following sections focus on: (1) strategies to improve the capacity and efficiency of photosynthetic C assimilation, (2) how changes in sucrose utilization when manipulating photosynthesis affect plant growth, (3) sucrose transport from the source to the sink organs, and (4) utilization of sucrose at the sink organs. We also summarized the impact of environmental cues on the translocation of sucrose to the sink and its utilization at the sink level. In addition, the strategic approaches involved in crop-yield improvement are discussed.

## 2. The Definition of Terminologies in Source-to-Sink Interaction

Manson and Maskell proposed “source and sink” in plants a few decades ago [7]. The terms “source production” and “sink utilization” of photo-assimilates are now frequently used in research related to plant resource allocation. “Source” tissue is the producer and exporter of photo-assimilates, while “sink” tissue is the importer and consumer of the assimilates [8]. Examples of source tissues of carbon (C) are fully expanded (older) leaves and green parts of plants, whereas sink tissues are roots, tubers, fruits, and seeds. Of all sink tissues, roots are the major putative source of inorganic nitrogen (N), whereas fully mature leaves are the main producers of organic nitrogen. The developing tubers/fruits/seeds serve as sinks for organic and inorganic N. Another term used in source–sink interaction is mass-flow, a transport system that links the source with the sink organ. In this context, flow refers to the xylem and phloem transport systems. The phloem sieve tube is involved in the movement of most organic nutrients basipetally to the sink organ, whereas the xylem transports nutrients from the sink to the source tissues [9].

## 3. Improving the Capacity and Efficiency of Photosynthetic Carbon Assimilation

Efficient photosynthetic carbon (C) assimilation is crucial for the overall yield of the reproductive sink organs [10]. One of the major bottlenecks of photosynthesis is the reaction of enzyme ribulose-1, 5-bisphosphate carboxylase/oxygenase (RuBisCO) with oxygen, leading to photorespiration, which reduces the efficiency of photosynthesis. RuBisCO has a slow catalytic rate of fixing CO_2_ with a turnover frequency of 3–10 per second [11] compared to most enzymes. Furthermore, RuBisCO demands a substantial amount of N and water from the plant, consuming 25% of N in a typical C3 plant. As such, reducing photorespiration is a prerequisite to improving photosynthetic efficiency, since improved photosynthetic efficiency is strongly related to water-use efficiency (WUE) and N-use efficiency (NUE). When the stomata open in C3 plants, CO_2_ moves in, and oxygen (O_2_) exits the cells. Upon closure of the stomata aperture under dry and harsh conditions, O_2_ accumulates to reduce the efficiency of the C3 pathway. Any component that indirectly influences stomatal conductance, such as root architecture, may increase water availability and promote C assimilation [12]. A reduction in stomatal resistance via the opening of the stomata aperture promotes higher CO_2_ uptake for photosynthetic C input at the expense of substantial water loss and adversely affects plants in semiarid or arid environments [13]. However, improving the conductance of CO_2_ from the substomatal spaces to Rubisco should possibly increase photosynthesis without adversely affecting water use [14]. Some other plants that have evolved adaptation to withstand harsh and dry conditions are C4 and crassulacean acid metabolism (CAM) plants. The C4 photosynthetic mechanism allows plants to pump CO_2_ into specialized cells in the bundle sheath cells to shield plants from the clusters of oxygen that might result from stomatal closure during dry conditions [14,15]. This allows C_4_ plants to withstand habitats that may be too harsh for C_3_ species because of their improved water- and nutrient-use efficiency [16,17]. Genetic approaches have also been established to pinpoint the anatomical development of C4 (Kranz anatomy), but the introduction of the functional C4 system has become a dilemma. If this is obtainable, the introduction of the C4 system into the ancestral C3 plants can minimize the amount of Rubisco and nitrogen fertilizer required and enhance water-use efficiency [16]. Nevertheless, plants without C4 evolution still undergo a highly rated selection potential for photosynthetic efficiency, nitrogen-use efficiency, and water-use efficiency with transgenic approaches [18].

Over the past four decades, studies have identified a broad range of strategic approaches to improve photosynthetic efficiency, as summarized in Table 1. These approaches are summarized into five distinct categories: (i) those focusing on a more catalytic version of RuBisCO and individual enzymes of the Calvin-Benson cycle [19,20], (ii) those engineering CO_2_-concentration mechanisms (CCMs) into the C3 photosynthetic pathway [21,22,23], (iii) strategies required to speed up the adaptation of the photosynthetic system under natural shaded or fluctuating light conditions by increasing the quantity of the photosystem subunit [24], (iv) those engineering synthetic bypass systems for photorespiration to optimize the uptake of CO_2_ [25], and (v) those that correlate the swiftness in stomatal response and photosynthetic C assimilation at the expense of water loss [26]. More recently, transgenic approaches have revealed that a reduction in stomatal aperture can increase WUE while enhancing C assimilation or crop yields [27]. Another photosynthetic carbon-improvement approach is to ensure that photosynthesis is less affected by feedback regulation when sinks lack the potential to fully utilize all the sugars supplied by the source leaves. Significant progress has been made in studying the extant of sink regulations for decades; a deeper insight into how sugar is sensed and the signaling pathway will be required to activate the signaling pathway and fine-tune the feedback regulation of photosynthesis. Another alternative way of alleviating photosynthetic feedback inhibition is by increasing photosynthate transport and utilization at the sink organs, which will be discussed in subsequent sections.

### 3.1. Impact of Global Warming on the Strategies to Improve Photosynthetic Carbon Assimilation

To meet the global food production demand, crop yields of most staple crops must increase by over 60–110% in the next 30 years [28]. At the same time, atmospheric CO_2_ has been envisaged to reach 550 ppm in 2050 [29], and this is usually accompanied by rising terrestrial air temperature, all in the name of global warming [30]. Today, the increasing threat of global warming is becoming more alarming, and the looming effect of these greenhouse gases can halt the positive yield prospect of our future crops. Thus, approaches to increase crop yields need to consider the future impact of global warming on crop yields. A more realistic approach to enhancing crop yields in the future is through improving photosynthetic carbon efficiency [31].

As mentioned above, various photosynthetic improvement strategies have been proposed (see Table 1), some of which have been examined under various climatic conditions, such as high atmospheric CO_2_ and temperature. Accelerating the rate of ribulose-1,5-bisphosphate (RuBP) regeneration [32,33] is a more promising strategy to offset the predicted climatic change [32,33]. With the current ambient atmospheric condition, C3 photosynthesis is limited by the carboxylation capacity of RuBisCO. However, it has been envisaged that in the future, elevated CO_2_ and higher temperatures will shift the leaf photosynthesis model of carbon uptake and assimilation [34] toward the regeneration capacity of RuBisCO [35]. In case there are no other changes, a higher temperature would accelerate the activity of RuBisCO while reducing its specificity for CO_2_ as compared to O_2_. However, this will narrow the range of internal CO_2_ under which RuBisCO is limiting and reduce the CO_2_ at which RuBP regeneration becomes limiting. In principle, the positive impact of the increased capacity for RuBP regeneration (which is one of the important improvement strategies) would therefore be greatest under both rising CO_2_ and elevated temperature conditions. With this progress, future crops will be well adapted to the forecasted high climatic conditions (elevated CO_2_ and temperature).

The overexpression of the redox-regulated Calvin cycle enzyme, sedoheptulose-1,7-bisphosphatase (SBPase), is another strategy used in improving photosynthesis and yield of *Arabidopsis* [36], tobacco [37,38], tomato [39], and wheat [40]. Based on the research progress made on these crops, Köhler et al. [41] further tested the impact of climatic change on the SBPase enzyme. They overexpressed cyanobacterial bifunctional fructose-1,6/sedoheptulose-1,7-bisphosphatase (FBPase/SBPase) in soybean (*Glycine max*) grown in the field during three growing seasons under rising CO_2_ of 600 ppm and elevated temperature of +3.4 °C. The resultant effect was further compared with normal ambient conditions (control). All the overexpressing lines had a significantly higher carbon assimilation rate across the treatments. Under ambient CO_2_, elevated temperature led to significant seed-yield reductions in both the control and the overexpressing genotypes. However, under elevated CO_2_ and increased temperature, the transgenic lines maintained higher seed-yield levels, while wild-type plants exhibited reduced seed yields compared with plants grown under elevated CO_2_ alone. These findings suggest that manipulating the photosynthetic carbon-reduction cycle could avert the proposed detrimental effects of future climatic change in plants. More studies should document the impact of global warming on strategies to improve carbon assimilation to ascertain future sustainability.

### 3.2. Photosynthetic Carbon Assimilation Contributes to Sustainable Development Goals

Imagine a world free of malnutrition and poverty, where people work to deliver shared prosperity in harmony with nature. This ideology moved society into declaring the future it wants, and the plans made were facilitated by the United Nations (UN) to launch the Sustainable Development Goals (SDGs). The UN 2030 agenda is a positive pathway to a sustainable livelihood, inclusive society, and sustainable environment. At the core of this agenda, there are 17 sustainable development goals [42], all of which must be globally achieved by the year 2030. Since photosynthesis is the underlying basis for greater crop-yield output [43], improving photosynthetic carbon assimilation is, therefore, a chief contributor to SDGs. Even though the objectives of the SDGs are intertwined, SDG 2, “end hunger, achieve food security and improved nutrition, and promote sustainable agriculture”, and SDG 13, “take urgent action to combat climate change and its impacts”, explicitly explain the contribution of improved carbon assimilation to achieving them.

Hunger is a global epidemic affecting underdeveloped, developing, and even developed nations. Globally, over 820 million people are severely food-insecure and malnourished [44]. In developed countries where food supply seems adequate, discrepancies in food prices add to the global hunger crisis due to increased food costs [45] and concerns of uncertainty. Furthermore, with the challenges of unemployment, high medical bills, and living expenses, millions of people are struggling to feed their families. Hence, in a bid to survive, people instead opt for cheaper undernourished foods to prevent starvation. It is imperative, therefore, to ask why the hunger crisis prevails.

Climate change plays an integral part in this global challenge, as it negates plant health, leading to drought, thereby limiting food production. Since plants have more potential to effectively resist atmospheric CO_2_ under ambient atmospheric CO_2_, increasing the number of plants will increase the absorption of atmospheric CO_2_. With recent research developments (Table 1), genetic engineering of CO_2_-concentration mechanisms (CCMs) into the plants and other strategies to improve carbon assimilation could be effective means to offset the impact of global warming on plants, ensuring food availability and sustainability. Facilitating these strategies could translate to greater yield output, consequently eradicating hunger, malnutrition, and poverty, thereby contributing directly to SDGs 1, 2, and 13. Although several studies have been conducted on photosynthetic-improvement strategies, including the genetic-engineering approach and RuBisCO regeneration, research efforts should be intensified on breeding plants that can readjust to the predicted 550 ppm CO_2_ in the future. If hunger could be eradicated and the impact of global warming combated, people will have access to basic nutrition, health, education, sustainable energy, and an inclusive society, among other benefits. Importantly, the implementation of this plan starts with us.

**Table 1:** Approaches employed to improve photosynthetic carbon assimilation in different plants.

Host Species	Strategies	Summary of Findings	References
C3 and C4 plants	Modification of in vitro assay method to measure the variability in carboxylase and decarboxylase activity of C_3_ and C_4_ leaf extract.	RuBisCO activation status is lower in mature C_4_ monocot leaves than in C_3_ monocots.	[19]
Rice	Model analysis conducted on both pot and experiments under various nitrogen rates.	Improved carboxylation rate due to higher RuBisCO content in mutant plants.	[46]
Tobacco	Expression of zeaxanthin and violaxanthin in the xanthophyll cycle coupled with an increased amount of the photosystem II subunit.	Greater than 15% increase in plant biomass.	[24]
Potato	Overexpression of pyrophosphatase in mesophyll cells.	Enhanced source and sink capacity and a doubling in starch yield of tuber.	[47]
Wheat	Overexpression of *Brachypodium distachyon* sedoheptulose-1,7-biphosphatase.	Increased leaf photosynthesis, biomass, and crop-yield potential.	[40]
Tobacco	Overexpression of *Arabidopsis* sedoheptulose-1,7-bisphosphatase (SBPase).	Improved photosynthetic capacity and crop yield.	[38]
*Arabidopsis*	Independent or synergetic alteration of sedoheptulose 1,7-bisphosphatase (SBPase), glycine decarboxylase H-protein (GDC-H) protein, and fructose 1,6-bisphophate aldolase (FBPA).	Enhanced carboxylation efficiency, vegetative biomass, and maximal seed-yield increase.	[36]
Potato	Expression of polyprotein comprising three subunits of *Escherichia coli* glycolate dehydrogenase (GlcDH).	High carbohydrate levels synthesized in the source leaves were utilized by the sink organ, facilitating a 2.3-fold increase in tuber yield.	[48]
*Arabidopsis*	Expression of a synthetic, light-gated K^+^ channel BLINK1 in guard cells surrounding stomatal pores.	BLINK1 facilitates a 2.2-fold increase in biomass in fluctuating light without the cost of water use by the plant.	[27]

## 4. Alterations in Carbohydrate Partitioning When Manipulating Photosynthesis Affect Plant Growth

Crop yields depend on photo-assimilates synthesized through photosynthesis (source capacity) and their utilization at the sink organ. Thus, experimental manipulations of source activity and sink strength explain the strong coordination between carbohydrate utilization at the sink level and photosynthesis. Photosynthetic products are primarily translocated to the sink organ in the form of sucrose (synthesized at the source leaves) for sink growth development. Thus, any activity that improves photosynthesis could also increase plant growth. In most cases, low sink activity resulting from sink removal or the integration of nutrient deficiency enhances carbohydrate accumulation in source leaves, and photosynthesis becomes downregulated [49,50]. A decline in photosynthesis could also occur when sucrose export from the source leaves to the sink organs becomes inhibited due to the downregulation of sucrose transporter genes [51]. In both cases of decreased sink activity or restrictions in sucrose transport, sucrose accumulates at the source leaves, increasing the expression of genes involved in carbohydrate storage and utilization, while repressing photosynthetic genes expression and final plant growth [52]. However, increases in sink demand have also been reported to improve photosynthetic activity and sink growth [53,54]. For example, 50% of blueberry plants were defoliated due to increased sink demand, but high photosynthetic capacity and yield were maintained [54]. Kaschuk et al. [53] have also shown that increased sink demand due to N_2_ fixation in soybean (*Glycine max*) relative to the nitrate-fed plants underpinned the observed increase in photosynthetic capacity and delayed leaf senescence. As such, plant breeders aiming at improving crop yield should be cognizant of the fruit pool size and photo-assimilate delivery from source leaves to sink organs (fruits).

The growth of plants at elevated CO_2_, which alters source supply, has revealed the strong association between source photosynthesis and sink demand. In most C3 plants, elevated CO_2_ directly enhances photosynthesis, which has led to increased carbohydrate supply in leaves, and an ultimate increase in crop yields [55]. Previous studies have shown that photosynthetic stimulation by elevated CO_2_ may be limited by the sink’s capacity to utilize or store additional photosynthates in C3 plants [56]. The initial increase in the photosynthetic rate was then greatly reduced by the suppression of photosynthetic activity due to the negative feedback caused by limited sink capacity. This is an indication of photosynthetic limitation by the sink after the initial limitation of carbon partitioning and growth by the source activity. Subsequent findings have revealed that the degree of photosynthetic stimulation by the interacting elevated CO_2_ and environmental, experimental, or genetic factors determines the sink strength [57,58]. For example, faba beans grown under an elevated CO_2_ (700 ppm) environment and exposed to well-watered versus drought treatments exhibited increased photosynthate accumulation in the leaves, leading to feedback inhibition of photosynthesis [58]. Similarly, poplar (*Populus* spp.) trees, which export more than 90% of photosynthate during the day, maintain high stimulation of photosynthesis at elevated CO_2_ [59]. Thus, maintaining the photosynthetic stimulation at elevated CO_2_ could be strongly associated with the capacity of the sink organ to utilize or reserve the additional carbohydrate [57,58].

Synthesis and degradation of starch is also a clear indication of carbon supply and utilization, especially in *Arabidopsis* [52,60]. The fixed carbon obtained during photosynthesis could be converted to sucrose (usually stored in the cytosol) and starch (majorly stored within the chloroplast of most plants) in the source leaves. In most plants, source leaves (older leaves) majorly export all the fixed carbon to sink leaves and other reproductive parts of plants for growth and other metabolic activities. Plants accumulate starch more rapidly during the day and degrade it at night to support growth and metabolism. The leftover starch is reserved until dawn. The rate of starch synthesis and degradation across a range of photoperiods is adjusted to avoid carbon starvation [60,61]. Weraduwage et al. [62] reasoned that the optimal partitioning of carbon to starch would occur when the carbon available for growth was constant day and night. As such, the budgeted carbon for growth should be the same during the day and at night because all the energy from growth is majorly attributed to this fixed carbon [61]. The growth derived for carbon usage may be more efficient than that obtained at night if ATP can directly optimize growth. On the other hand, the carbon budget for growth at night could also be greater than that during the day because sucrose synthesized during the day requires one ATP plus the energy demand in the tricarboxylic cycle. However, carbon primarily stored as starch and then transformed to sucrose requires one and two-thirds (assuming two-thirds of the nighttime export is maltose and one-third is glucose) [62]. Thus, the photosynthetic products are strictly adjusted based on the plants’ demand. Interestingly, increase in starch content at the end of the light period (which supports carbon metabolism and growth at night) decreased with biomass accumulation across 94 *Arabidopsis* accessions [63]. This indicates that plants with a strong affinity for starch accumulation exhibit reduced growth, while plants with improved growth rates incur better carbon-use efficiency, re-emphasizing the relationship between photosynthetic machinery and plant growth.

## 5. Transport of Sucrose to the Sink

The need for a high C assimilation rate elucidated above can only be effective if the increased C supply can be utilized by downstream transport and plant metabolic processes. Thus, all photo-assimilates not required for leaf function are turned into sucrose or amino acids and translocated to the sink organ via the phloem [2]. Up to 80% of photosynthetically fixed carbon can be exported from mature leaves. However, the quantity of sucrose available for export from source leaves anchors on factors including photosynthetic activity (primarily the carbon-fixation), partitioning between the starch synthesis within the chloroplast and triose-phosphates moved out of chloroplast for sucrose synthesis, and temporary accumulation of sucrose in the vacuole [64]. The synthesized sucrose is then exported to various plant organs depending on demand, while other redundant sucrose is converted into starch and stored for further utilization. Alteration in any of these aforementioned factors can affect the quantity of sucrose available for export, thereby changing the source–sink balance [64].

**Figure 2:**
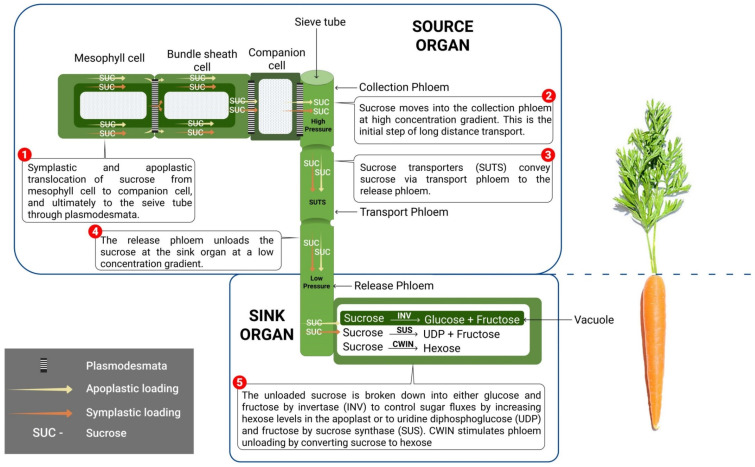
Schematic diagram of symplastic and apoplastic transport of sugar from source to sink organ.

Photo-assimilate transport comprises short-distance (e.g., loading/unloading of sucrose) and long-distance transport [65]. Phloem cells that convey organic materials and sugars within the plant are called sieve elements. The known mechanisms of active phloem loading presently are: (i) symplasmic loading, where de novo-synthesized sucrose has to exit the mesophyll cells and transfer from cell to cell via plasmodesmata (PD) (which acts as the bridge across the cell walls) into the sieve elements (Figure 2); (ii) apoplastic loading; which involves the movement of sucrose from mesophyll cells to companion cells against a concentration gradient (Figure 2); and (iii) “polymer trapping”, which involves the conversion of sucrose into larger sugar polymers such as stachyose, verbascose or raffinose symplasmically supplied by intermediate cells [64,66]. All these aforementioned pathways follow the three phloem sections (collection, transport, and release phloem) to effectively unload sucrose at the sink organ. These entail sucrose loading into the collection phloem (embedded at the leaf blade) [67], which signifies the first step of long-distance transport (Figure 2). The subsequent step is the transport of sucrose via the transport phloem (path of sucrose transport) connecting the source leaves with sink organs, and then the final delivery of sucrose via the release phloem to the sink organs (Figure 2) [64,67].

In various loading-pathway routes, specific transporters are needed to efficiently transport sucrose across plasma or within the intracellular compartment. Thus, these sucrose transporters serve as significant regulators of sugar flux and accumulation [68,69]. These transporters are localized in the three phloem sections, and their function cannot be underestimated, especially in tree crops where sucrose loading is symplastic in the collection phloem [70,71]. Nevertheless, sucrose is removed from the phloem via either a symplastic or an apoplastic pathway in the release phloem, albeit the preliminary steps are often symplastic [72]. Thus, disruption in the symplastic pathways in sink organs like developing seeds requires an apoplastic step to efficiently transport photo-assimilates. It is noteworthy to mention that regulating these sucrose transporter genes significantly improve crop yields [69]. A good example is the sucrose transporter (*OsSUT2*), which functions in the transport of sucrose from the vacuole across the tonoplast. Disruption in the function of the *OsSUT2* transporter restrains sugar transport basipetally from source leaves to sink organs; thus causing major physiological disorder and rice yield loss [73]. This indicates the crucial roles of sucrose transporters displayed in phloem loading and unloading at the sink organ. During the early stages of the tuber developmental stage in potato, tuber-specific inhibition of *SUT*1 reduces the fresh weight, demonstrating the potential role of *SUT*1 in phloem unloading [9]. More research attention have been directed towards a new class of sugar transporters, Sugars Will Eventually be Exported Transporters (SWEETs), in *Arabidopsis AtSWEET*10–15 [74], and rice *OsSWEET* 11 and 14 [75] which are responsible for sucrose export from the transport phloem to the apoplast. The *Arabidopsis* double mutant, *atsweet11* or *12*, exhibits impaired ability to expunge sucrose out of the leaves. This inhibition resulting from starch accumulation leads to downregulation of photosynthesis, demonstrating that sucrose export by the SWEET family plays an imperative role in photosynthetic improvement. Sucrose unloaded into the apoplastic space is assimilated by the sink cells or bonded by invertase to hexose transported by specific carriers (Figure 2). As such, sucrose can either be stored for sink growth and development or left in the vacuole of the storage cells of some crop species, such as sugar cane and sugar beet [76].

## 6. Sucrose Utilization at the Sink

Photo-assimilates are symplasmically unloaded from the phloem through plasmodesmata (PD), which serves as a bridge connecting the surrounding cells to sink organs [68]. The sucrose delivery by the release phloem (Figure 2) improves physiological growth of sink organs. The homogenous distribution of assimilates within the sinks is a major driver of plant productivity, and it is expressed as the harvest index (HI). The HI is calculated as the proportion of harvested dry weight divided by overall plant dry weight or above-ground dry weight. Thus, plants with high HI have a greater percentage of its photo-assimilates diverted back to the sink [64].

There exist fluctuations in carbon partitioning and shifts between the symplastic and apoplastic pathways throughout plant developmental processes. The pathway through which sucrose is unloaded depends on the specific sink involved and its developmental stages [77,78]. For example, sucrose unloading is apoplastic during the fruit developmental stage in apple [79]. In potato [80] and white mature jujube [77], the mode of phloem unloading transits from apoplastic to symplastic. Contrary to this, sucrose unloading is symplastic during the early and middle stages of grapefruit development, but subsequently switches to apoplastic during the fruiting stage [81]. The switch to a symplasmic unloading pathway route is mainly based on the amount of plasmodesmata [77], an indication that symplasmic unloading strongly exceeds the transport capacity of the apoplastic pathway. At the sink level, sucrose is broken down into glucose and fructose by invertase (INV) [82] to control sugar fluxes by increasing apoplasmic levels of hexoses in the apoplast (Figure 2). Sucrose can also be degraded into uridine diphosphoglucose (UDPG) and fructose by sucrose synthase (SUS), albeit the energy demand for degradation is higher in INV than SUS. INV is encoded in cell-wall invertase (CWIN), which plays an imperative role in apoplasmic phloem unloading in sink organs. CWIN-derived glucose predominates the sink tissues to trigger cell division during early seed development, whereas sucrose synthase synthesizes starch, cellulose, and protein during the late developmental stages for sink strength [83,84]. Cell-wall invertase also increases sucrose unloading to the sink organ by converting sucrose to hexoses (Figure 2). Since plants are highly susceptible to sucrose imbalance in the sink organ, promoting the activity of endogenous CWIN by silencing its inhibitor is a potential molecular strategy to lessen the effect of sink abortion.

## 7. Influence of Environmental Factors on Photo-Assimilate Transport

Plants undergo a wide range of environmental changes all through their life-cycle, and have evolved diverse strategies to withstand such changes. The impact of environmental factors on photo-assimilate transport within the source and sink is discussed in the subsequent sections.

### 7.1. Effects of Carbon Dioxide

**Figure 3:**
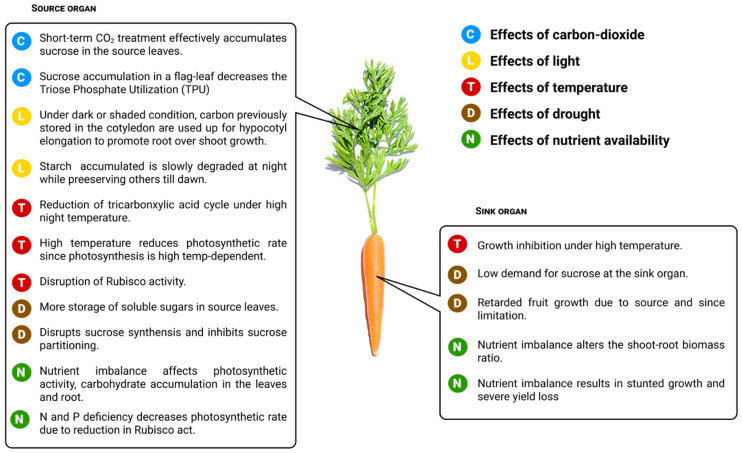
The impact of environmental factors on photo-assimilate transport.

Nowadays, the atmospheric concentration of carbon dioxide (CO_2_) is approximately twice that which prevailed over the past few centuries [1] due to the impact of fossil combustion and inappropriate use of agricultural lands. Indeed, the concentration of CO_2_ in the atmosphere increased from approximately 315 ppm a few decades ago to an average of 390 ppm in these present days. The concentration has been envisaged to be within the range of 540 to 970 ppm before the end of the century. Despite the pressure imposed by this increased atmospheric CO_2_ on the global climate, its impact on plant photosynthesis cannot be underestimated. A rise in CO_2_ could promote photosynthetic rates and carbohydrate production, and positively affect phloem transport and growth. However, these factors depend on either the short- or long-term CO_2_ enrichment terms. Most plants subjected to short-term CO_2_ treatment effectively accumulate carbohydrates in the leaves, increasing biomass partitioning between their source and sink organs (Figure 3). However, prolonged exposure (long-term effects) of some other plant species to elevated CO_2_ concentration reduces the initial stimulation of photosynthesis and decreases the photosynthetic rate [85]. For example, an increase in the CO_2_ concentration of soybean plants subjected to 27 days of treatment from 400 to 1000 ppm resulted in a drastic reduction in photosynthetic rate [86,87], which subsequently affected the crop yield. This reduced net photosynthetic rate may be due to the reduced concentration and activity of RuBisCO [88] or the source–sink imbalance emanating from leaf carbohydrate accumulation under increased CO_2_ concentration [89]. This reduction in the net photosynthetic rate observed during long-term exposure could also be advantageous, as it potentially enhances the remobilization of assimilates from the source leaves to the sink for proper readjustment of the source–sink balance [90].

Source–sink imbalance is mostly apparent during plant exposure to elevated CO_2,_ when photosynthetic rate outweighs the transport capacity or the capacity of sinks to utilize assimilates for growth. This incapability results in carbohydrate accumulation in photosynthetically source leaves [90]. Contrary to this opinion, CAM plant *Opuntia ficus-indica* exposed to long-term high CO_2_ concentration was not affected by the photosynthetic rate [91]. In fact, after three months of subjecting the plant to double CO_2_ concentration of 750 mol/mol, the glucose, starch, and malate contents in the basal cladodes increased significantly compared with the low CO_2_ concentration of 370 mol/mol; however, the sucrose content was not affected [91]. The sucrose content in mother cladodes was not affected because it was translocated to daughter cladodes by an active phloem transport, thereby resulting in a marked increase in the biomass of daughter cladode after three months of exposure to high CO_2_ concentration [87,91]. The impacts of elevated CO_2_ on photosynthetic carbon assimilation are not the same, since some plant species are easily affected by elevated CO_2_ than other plant species. Thus, variations in plant species response to elevated CO_2_ are mainly engendered by diverse plant photosynthetic types.

Triose phosphate utilization (TPU) explains the response of photosynthesis to carbon source–sink imbalances at elevated CO_2_. Although several studies have shown that elevated CO_2_ treatment [92,93,94,95], experimental manipulation (pruning) of source–sink carbon [96,97], or a synergetic impact of both can influence source–sink balance [98,99], more exciting advances have recently emerged. Fabre et al. [100] explored the impact of source–sink imbalances on photosynthesis, which is best predicted by the limitation of triose phosphate utilization. They also investigated the response of TPU on photosynthetic regulation under such imbalances. The group hypothesized that there is a linear relationship between the source–sink ratio and TPU limitation in rice leaves. To test this hypothesis, they subjected rice plant to elevated CO_2_ in order to achieve increased source capacity, and also pruned the panicle (sink) to decrease the sink. They observed a genuine association between the source–sink balance and TPU capacity. There was a negative correlation between TPU capacity and the flag-leaf sucrose concentration (increase in sucrose decreases the TPU), and a linear correlation between TPU and photosynthesis (photosynthesis decreased during the day along with TPU) (Figure 3). This reduction in TPU is associated with the accumulation of sucrose in the flag-leaf resulting from sink limitation. As such, TPU could be adjusted to be slightly higher than the photosynthetic rate or vice versa, albeit the case might be different in plants whose sink adjustment and assimilate transport to increased assimilation potential is poor.

### 7.2. Effects of Light

Light is one of the most paramount factors affecting shoot and root development during early seedling development [101]. Energy derived from light facilitates carbohydrate synthesis during photosynthesis. When seedlings are kept in the dark or shaded from light at the early germination stage, a larger quota of the C assimilate previously stored in the cotyledons are used up for hypocotyl elongation (Figure 3), inhibiting the root growth. Growing seedlings under the shade with a low ratio of red to far-red light induces hypocotyl elongation to promote shoot over root growth (Figure 3) [101]. This occurrence moves cotyledons, as well as the shoot apex, toward the light. Subsequently, the de-etiolated seedling begins photosynthesizing to produce sucrose, since the delivery of assimilate to the root is subject to changes in sucrose availability. Studies have revealed that photosynthetic transport of sucrose from the shoot (cotyledon) to the root functions as a direct signal to activate and enhance root elongation of *Arabidopsis* in a light-dependent manner [4]. This finding and other related findings [102,103] support the efficacy of carbon resources in coordinating long-distance transport in the presence of light, as the illumination of shoot promotes root growth via shoot-to-root signaling.

To cope with nights characterized by lack of sunlight and prevailing darkness, plants devised a coping strategy similar to that of a battery. During the day, a portion of plant photosynthates is allocated for storage in the form of starch. At night, in the absence of light and photosynthesis, these stored resources are reallocated to sustain plant growth and metabolism (Figure 3) [104,105]. Starch degradation at night clearly explains the carbon economy of the plant, as well as plant growth. Starch degradation, carbon availability, and growth at night progressively decrease as the photoperiod becomes shortened [60]. Plant growth rate anchors majorly on the amount of starch available at dusk and the length of the dark periods [60,106]. In short photoperiods, starch is mostly exhausted at dusk due to carbon limitation in plants during this period, causing severe growth inhibition [107,108,109]. If the duration of the night is suddenly extended, starch will be completely consumed within 1 to 2 h, the amount of sucrose becomes drastically reduced, catabolism will be activated, and ultimately growth becomes inhibited. As such, proper coordination of starch degradation and growth is an expedient to preventing either the accumulation of sugar or its depletion to a point where catabolism is activated. This coordination could be obtainable if: (1) the circadian clock acts directly to regulate plant growth rate and timing, and (2) the coordination could also occur through an indirect action of the clock by regulating the rate of starch degradation and determining the amount of sucrose available for growth [109]. Unlike the short photoperiod, during which growth decreases in the early hours of the day, plants subjected to longer photoperiods exhibit increased growth during the night and at the start of the day. There is a more pronounced lag before starch accumulation commences again [60]. The delay in starch accumulation is mostly accompanied by increased sucrose synthesis and other soluble metabolites, indicating that growth is not carbon-limited. Future research should focus on the cause of this delay; this could have been due to limitations arising from starch accumulation or introduction of degraded starch towards the end of the light period.

Light also regulates phloem loading of sucrose from the photosynthesizing leaves, either “apoplastically” or “symplastically” [110,111]. Apoplastic loaders (pea and spinach) and symplastic loaders (pumpkin and *Verbascum phoeniceum*) acclimatize to photosynthesis differentially in response to light environment. Thus, increased cell-wall invagination, indicating no greater starch levels, was observed in the leaves of apoplastic loaders transferred from low to high light condition [112]. However, in symplastic loaders, there was no increase in the plasmodesmatal frequencies per loading cell, thereby accumulating sugar in the source leaves [112,113]. This modification is consistent with the limitation in carbon export capacity to increase plasmodesmata density in mature leaves.

### 7.3. Effects of Temperature

Extremes of temperature are harmful to plant health. Thus, maintaining a favorable leaf temperature is essential for plant growth because photosynthesis can be maximized within a relatively narrow temperature range [1]. Most biochemical reactions of photosynthesis are disrupted (Figure 3), since photosynthesis is a temperature-dependent process [114]. Temperature also affects the membrane integrity of the chloroplast [1] and irreversibly disrupts RuBisCO activity (Figure 3) [115]. Recently, cocoa seedlings subjected to an elevated temperature of approximately 39 °C heat treatment against 36 °C in chamber control were negatively affected by the photosynthetic rate and biomass accumulation [116]. In addition, wheat plants subjected to heat stress also exhibited disruption in floret settings, resulting from limitation in photo-assimilate supply at the source leaves and varying photoperiodic sensitivity [117]. This implies that photo-assimilate supply and the quantity of reserved assimilate stored in the vegetative tissue are key determinants of floret formation and subsequent grain (sink) development under heat stress [118,119]. Although ample evidence exists regarding the negative impact of heat stress on crop yields [120,121,122], little is known about the source–sink metabolite dynamics and crop-yield interaction [123]. More recently, Impa et al. [124] examined the metabolic changes in six winter wheat genotypes by investigating the post-heading high night temperature (HNT) effect on carbon balance, sink–source metabolic changes, and other yield-related traits relative to the control experiment (at 15 °C minimum night temperature). In the same study, a marked increase in proteinogenic amino acids and carbohydrates, such as sucrose, glucose, fructose, and raffinose, was detected in the spikes (sinks) during HNT relative to the control condition. However, a drastic reduction of the tricarboxylic acid cycle intermediate compounds was found in the source leaves (Figure 3). To this end, changes in carbohydrate metabolism and tricarboxylic acid cycle intermediate in the spikes and leaves provide insight into metabolites involved in HNT response [124]. Wang et al. [125] investigated the effects of heat stress on 38 wheat varieties (with different levels of thermo-tolerance) to unravel the proteomic and metabolomic changes induced by this stress during the grain-filling stage. They observed a marked increase in the free amino acid levels and a drastic reduction in the content of carbohydrate metabolism and tricarboxylic acid (TCA)-related metabolite in response to heat stress. This result suggested a plausible wide range of mechanism for heat-adaptive metabolism required to maintain the grain-filling process in plants [125]. Plant breeders should focus on the integration of this metabolomic and other omic approaches; this could provide an insight into the appropriate markers that can be used to address the issue of climate change in this century.

Low temperature also poses a major threat to sugar transport within different cell types (intermediary cells, parenchyma transfer cells, sieve elements) in diverse manners. Plants with a symplastic minor-vein configuration seem to be dominant in tropical and sub-tropical regions, whereas plant species with an apoplastic configuration are mostly found in temperate zones. Hence, one would believe that the symplastic loaders can be more cold-sensitive than apoplastic loaders [64]. The sensitivity of herbaceous species and deciduous trees with symplastic phloem-loading to cold was due to the collapse of intermediary cells under low temperatures, eliminating photo-assimilate transport and starch accumulation in the chloroplast [113]. These ultrastructural changes have also been found in broad leaf evergreen species *(Ajugareptans*, *Aucubajaponica*, and *Hederahelix*) that have a symplastic phloem-loading mode. At low temperature, leaves of this latter plant exhibit a higher exudation rate, but neither shows any symptoms of frost injury nor starch accumulation in the chloroplasts. Therefore, removing redundant photo-assimilates from cold-acclimated source leaves might be crucial to maintaining the functional and structural integrity of the plant [126]. However, physiological studies [127] showed no significant variation between symplastically and apoplastically phloem-loading species in response to cold, since the carbohydrate available for export in phloem-loading modes of both species was closely related. This has led to the hypothesis that there is a relationship between the phloem-loading mode and the growth architecture instead of the geographical distribution of both species.

Although low temperature drastically reduces plant growth rate and final biomass, even in plants well acclimated to low-temperature regimes, some geophytes still exhibit increased growth rate and a much larger storage organ under such a temperature regime [128]. This observed positive growth effect in storage organs was correlated with enhanced leaf longevity and extensive periods of carbon assimilation, which is partly related to improved biomass in the storage organ [128]. In the same study [128], higher temperatures halted corm (sink) growth (Figure 3) even before the first visual signs of leaf senescence became apparent, suggesting the great influence of sink activity on leaf longevity in spring ephemerals (*Crocus vernus*). As such, leaf senescence is induced by sink limitation once the carbohydrate reserves are replenished [129]. It can be inferred that the larger storage organs could have resulted from increased overall sink strength at low temperature gradients in these species. The production of a larger storage organ under a lower-growth temperature regime (8/6 °C) was also affirmed in *Erythronium americanum*. The increased growth rate observed at lower temperature gradients could be attributed to the delay in starch accumulation, leading to improved source–sink balance and delayed leaf senescence [130].

### 7.4. Effects of Drought

Considerable proportions of our global agricultural lands are prone to drought. Drought has been extensively reported to directly facilitate a wide range of yield-reduction symptoms in plants. These symptoms include photosynthetic inhibition [131], physiological metabolic disorders [132], and increased oxidative stress [133]. In a bid to survive, plants have evolved a series of morphological and physiological adaptive mechanisms to withstand a water-deficit condition [134]. When a plant root directly senses a water deficit, the biomass allocated to the root increases over that allocated to the shoot, facilitating increased root-over-shoot growth in rice [134,135], *Arabidopsis thaliana* [136], wheat [137], and soybean [138]. This case of plants’ response to drought is an indication that a higher root-to-shoot ratio could be an adaptive parameter to improve plants’ tolerance to drought stress [139]. Hence, maintaining an efficient root (sink) system is a precursor to increasing water uptake while preventing water loss in response to drought stress.

Excessive transpiration disrupts the synthesis of photosynthetic products (Figure 3), inhibits carbon utilization at different sink tissues [113]. Most research has documented that most drought-induced sugar metabolism and phloem loading alters carbohydrate levels in leaves at different plant developmental stages [140,141]. Plants subjected to drought stress usually have accumulated soluble sugars, including sucrose, stored in their source leaves (Figure 3). The accumulation of this stored sucrose in the source leaves act as an imperative energy derivative strategy which improves plant tolerance to drought stress condition [142]. Interestingly, sucrose could also be accumulated in the leaves if there is a low demand for sucrose at the sink cell [136]. Thus, sucrose accumulation in the leaves could be drought-induced or result from low-sink demand (Figure 3).

Drought stress also stimulates leaf senescence and enhances reserve remobilization, since these play an integral role in plant development and other fundamental strategies required for stress mitigation [143,144]. The concentration of sugar can also influence leaf development, with senescence being the fundamental causal signal and the primary substrate responsible for carbon remobilization to alleviate the drought-stress effect [145]. Transgenic tomato plants overexpressing *Arabidopsis* hexokinase (*HXK*) exhibit increased sugar contents, which suppresses photosynthetic activity and increases leaf senescence [146]. Meanwhile, drought-induced leaf senescence promotes the redistribution of assimilates to developing grains and increases rice grain-filling rate [147]. For instance, water deficit during grain-filling decreases seed size in soybean due to the reduction in the period of grain-filling [148,149]. Seed growth anchors majorly on the amount of assimilate supplied from the maternal plant (source activity) and the assimilate demand within the embryonic tissues (sink activity), indicating the pivotal role of both source and sink activity in enhancing seed growth under a drought condition. Thus, Westgate et al. [148] hypothesized that a rapid reduction of sucrose within the embryo depicts source limitation, whereas a delay in sucrose uptake connotes sink limitation. Despite the reduction in sucrose concentration caused by water deficit, the dry weight of the seed increases at or close to the control rate, thereby triggering the remobilization of reserve carbohydrates from all source organs to improve seed growth [148]. Drought could also impede fruit growth and development caused by both sink and source limitations (Figure 3) [150]. A well-ripened grape berry under drought is an excellent example of a sugar sink that exhibits a great yield reduction while increasing the total sugar content of the remaining berries [64]. The most drought-sensitive stage is the early developmental stage of grape berry; however, drought has no effect on sugar accumulation at this stage. This is an indication that sink strength within each of these berries is determined majorly by sink activity, rather than the berry size resulting from sugar accumulation [151]. For efficient crop-yield improvement, an in-depth understanding of the effect of drought on sugar accumulation at that early developmental stage (crucial stage) is required.

### 7.5. Effects of Nutrient Availability

Humans respond to nutrient deficiency, becoming malnourished under such conditions. Plants are not left out of this; any shortage in nutrient supply can dramatically affect resource allocation, resulting in stunted growth and severe yield loss (Figure 3). The effects of nutrient starvation on long-distance transport and assimilate allocation within plant tissues have been extensively studied [152]. Hu et al. [153] suggested that nutrient starvation can greatly influence the distribution of photo-assimilates directly through phloem loading and transport, or indirectly by reducing sink demand. Hossain et al. [154] also reported that the supply or deprivation of macronutrients affects the root-to-shoot partitioning of dry matter, especially in higher plants. All these findings point toward the imperative role of mineral nutrients in biomass reallocation and crop-yield improvement. Nutrient imbalance affects photosynthetic activity and carbohydrate accumulation in the leaves and root, and alters the shoot-to-root biomass ratio (Figure 3).

The efficient use of nitrogen (N) has been a bottleneck affecting global agricultural systems. N deficiency drastically reduces the photosynthetic rate, the number of flowers, and crop yields due to the decrease in the amount of RuBisCO protein and activity [155,156]. Roots subjected to N deficiency accumulate more photosynthetic products such as sucrose and sorbitol. Thus, unloading of photosynthates to the roots primarily serves as an energy source and signal molecule involved in root growth, thereby increasing the root-to-shoot biomass ratio [155]. Increased root carbon boosts the chances of the root to forage for more N in the soil or nutrient solution, indicating the relationship between the signaling roles of sugar and nitrate (NO_3_^−^) in improving shoot and root growth. However, plants subjected to low N concentration exhibit a reduced growth rate due to shortage in the supply of amino acid pool needed to sustain protein synthesis essential for the formation of new tissues. As such, the starch content in the leaves increases significantly. However, the mechanism behind this sugar accumulation in response to the deficit is yet to be clarified. Taken together, N-deficiency could result in biomass reduction, starch accumulation in the leaves, and greater carbon reallocation to the root, leading to increased root-over-shoot growth.

On the other hand, excessive N supply is mainly characterized by a wide range of ammonium (NH_4_^+^) toxicity symptoms, including ion imbalance, leaf chlorosis, pH regulation disorder, and physiological metabolic disorder [157]. The excessive N-replete also affects the net carbon assimilation rate via its effects on the photosynthetic component [158]. Studies have also reported that excessive N supply reduces the root carbon by diverting unassimilated NO_3_^−^ acropetally back to the shoot via xylem to promote shoot development, thus retaining more photosynthetic carbon in the shoot [159,160]. The development in the shoot organ only stimulates vegetative growth, reducing the number of flowers and fruit yield. This suggests that a balanced distribution of sucrose between the source leaves and flowers might act as major yield-determinant factors [158], since the flowering process depends on sucrose supply. In contrast to the findings regarding lower root:shoot ratio, a higher root:shoot ratio was observed under excessive NH_4_^+^ compared with NO_3_^−^ in tobacco [161] and cucumber [162]. The shift in biomass partitioning favoring the root implied accelerated phloem transport of assimilates to the root under NH_4_^+^ nutrition. The dynamic changes in root:shoot biomass ratio observed in most of these plant species resulted from the alteration in carbon partitioning between the root and shoot when different N rates were applied.

Besides nitrogen, phosphorus (P) is the second macronutrient required for plant growth and yield. P deficit directly affects photosynthesis due to the inorganic phosphate (Pi) availability in the chloroplast, resulting in reduced carbon assimilation in the leaves. Similar to N deficiency, phosphorus limitation induces carbohydrate accumulation, increasing the root:shoot ratio. Sucrose transport across the phloem is often stable and sometimes increases during the early phosphorus-deficit phases [163]. The significance of sucrose transport in P-deficiency signaling has been clearly demonstrated in white lupin [164]. Liu and colleagues argued that the expression of white lupin phosphate transporter, *LaPTI*, and a secreted acid phosphatase gene, *LaSAP1*, responsible for phosphate acquisition, is rapidly induced by phosphate deficit. Phosphate-deficient plants were then treated by stem-girdling to hinder shoot–root sucrose transport, but no induction of either *LaPT1* or *LaSAP* was observed. As such, the amount of sucrose in the leaf translocated to the roots was reduced by 95% in stem-girdled plants, indicating the importance of sucrose transport in plants subjected to phosphate-starved condition. However, the response of nitrogen (N) and phosphorus (P) deficiency seems similar due to the starch accumulation in the leaves and carbon sequestration in the root, increasing the root-to-shoot biomass ratio. However, N and P limitations are shaped differently in response to plant growth, since both macronutrients perform different functions in the machinery of plant energy metabolism, photosynthesis, and respiration [165].

Potassium (K^+^) is another macronutrient whose availability also affects the source-to-sink relationship. Potassium deficiency reduces the photosynthetic rate and plant growth due to the sucrose sequestration in the leaves [166,167]. One would think that increased sugar concentration in the leaves could enhance root sugar content; instead, root sugar and growth are disrupted, since sugar translocation is halted due to higher sugar content in the leaves. Hence, sucrose and starch concentrations in K-depleted plant roots are significantly reduced compared with the K-repleted counterpart [166]. A good explanation for this is that the reduction in sucrose exported to the K-deficient root is attributed to the changes in the concentration of K^+^, which could affect phloem-loading of sucrose [168]. A member of *Arabidopsis* potassium-transporter family, AKT2/3, has been identified as the photosynthate-induced phloem potassium channel, which affects sucrose-loading and long-distance transport by regulating the activity of sucrose/H^+^ symporter via the phloem potential [169,170]. The authors revealed that a significant fraction of net photosynthetic CO_2_ leaking out of sieve tubes seemed not to be effectively loaded (retrieval) into the phloem of the mutant. Hence, *akt2/3* exhibits a drastic reduction in the amount of sucrose in the phloem sap. These scenarios reduce the root-over-shoot biomass ratio of K-depleted plants.

Studies have also reported the influence of magnesium (Mg^2+^) on plant metabolic processes and reactions, including chlorophyll formation, photo-assimilate distribution, phloem-loading, photosynthetic carbon fixation, and partitioning [171]. Deficiency in magnesium increases the concentrations of sugars in leaves and reduces sucrose export to the root [172,173]. This reduced sucrose transport could either be due to impaired phloem-loading caused by reduced Mg-ATP availability [174] or by reduced metabolic activities of sink organs [175]. Hence, a high shoot-to-root biomass ratio is plausible. Although the allocation of carbon to the youngest leaves appears to be more dominant than to the root [176,177] yet, higher root-over-shoot growth is observed in certain species subjected to low Mg condition [177]. As such, Mg starvation reduces the growth of younger leaves compared with that of the roots. The early accumulation of sugars in Mg-deficient leaves results in the downregulation of genes encoding the chlorophyll a/b binding protein, *Cab2*, which is actively involved in photosynthesis [177]. Another gene encoding companion cell sucrose/H^+^ symporter, *BvSUT1*, expressed at the topmost part of the Mg-deficient beet leaves, had no effect on phloem-loading [177]. N- and P-starved plants have exhibited increased carbohydrate transport, but K and Mg deficiency is still exempted [163]. More studies should be conducted on the underlying mechanisms involved, as this is poorly understood. These findings depict the influence of mineral nutrient deficiency or supply on the assimilate distribution within the plant organs.

## 8. Functional Roles of Sugar Transporters in Mitigating Environmental Stress

Sugar transport plays a pivotal role in the regulation of plant growth and plant response to environmental stress [64]. Efficient transport of sucrose through the apoplastic pathway in the phloem depends on the involvement of plant sucrose transporters (SUTs) [74,178]. As discussed earlier, a considerable number of soluble sugars and sucrose-specific transporters have been identified, including the recently identified family of Sugars Will Eventually be Exported Transporters (SWEETs). SWEETs are integral membrane proteins characterized by seven *α*-helical transmembrane (TM) domains and two MtN3/saliva motifs [179]. The MtN3/Saliva motif is conserved in various organisms, with the proteins harboring this motif functioning as the sugar transporters [74,180].

**Figure 4:**
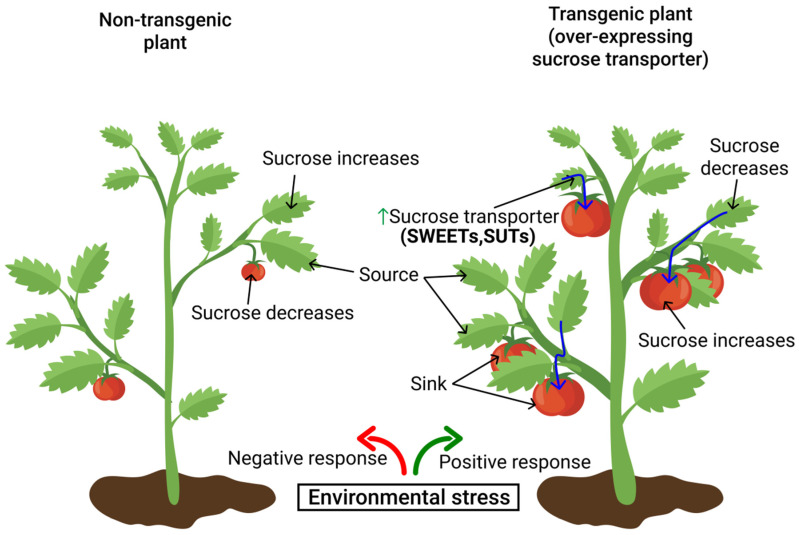
Sugar transporters are involved in environmental stress mitigation. Under stressed conditions, overexpression of sugar transporters (SWEETs, SUTs/SUCs) in plants (**right**) enhances the transport of soluble sugars, such as sucrose from source leaves to the sink organs for fruit development. Meanwhile, under the same conditions, increased sucrose accumulation in the leaves of nontransgenic plants (**left**) inhibits sucrose export from the source leaves, limiting sink (fruit) growth and development.

SWEETs regulate the redistribution of sucrose to the sink under adverse environmental cues, such as drought [181], cold-temperature stress, and elevated CO_2_ (Figure 4). *Arabidopsis thaliana* subjected to drought stress exhibited increased sucrose transport to the root when sucrose transporter genes, including *AtSWEET11*, *AtSWEET12*, and *AtSUC2*, are upregulated (Figure 4) [136,181] to rescue the sucrose lost to the apoplast. Similar findings revealed that the sucrose transporter gene *OsSUT2* was upregulated in the leaves of rice subjected to drought treatments [182], as enhancing sucrose transport from source leaves to the sink cells necessitates the cellular energy demands of the plant. More recently, the sucrose transporter genes *GmSUC2*, *GmSWEET6*, and *GmSWEET15* were upregulated in the leaves and roots of soybean seedlings subjected to drought stress, albeit all these upregulations plummeted under prolonged drought. This is an indication of increased capacity of sucrose unloading into seeds and activation of sucrose metabolism during the early seed developmental stage under a drought-stress condition [138]. However, during late seed-filling stages, basipetal sucrose flow from source leaves to seed decreased, leading to an impaired supply of seed (sink) metabolic need, and thus reduced seed weight or yield [142]. Since these transporters trigger sucrose export from source leaves to the root (sink), especially during the early plant-developmental stage, enhancing sucrose transport to the root is promising to optimize root development under drought stress [138]. Rooting depth is an essential trait for determining drought resistance in plants [183,184]. To this end, identifying sugar-transporter genes that enhance root-tip elongation can also play an important role in crop-yield improvement. Voothuluru et al. [185] conducted a proteomic analysis on maize root tips subjected to water deficit. The crew [185] indicated a strong interaction between *ZmSUT2* (localized in the tonoplast) and root elongation, albeit the functional role of this *ZmSUT2* transporter in mitigating drought is still unknown. Understanding its functional role will provide an insight into its contribution toward vacuolar-reserve remobilization, since the storage of sugars in the vacuole is imperative for osmotic adjustment under stressed conditions [186,187].

SWEETs also enhance cold tolerance in the tea plant. Wang et al. [188] demonstrated that the increased tolerance of the tea plant (*Camellia sinensis*) to cold in *CsSWEET16*-overexpressing (OE) lines were due to the combination of different levels of modified sugars. They affirmed that overexpressing *CsSWEET16* in *atsweet16-1* rescued the high fructose content in the mutant. Similar findings obtained from *AtSWEET16* explained the function of *CsSWEET16* in altering plant fructose content with an extended effect on glucose and other soluble sugar contents to improve plant tolerance to cold [189]. Under extremes of low temperature (cold), *CsSWEET16* OE lines exhibit reduced fructose accumulation in tea leaves, an indication that *CsSWEET16* participates in fructose export out of the vacuole. The reduced fructose content under such conditions could have been due to feedback regulation of sugar metabolism, which might affect glucose and other carbohydrates associated with cold tolerance. This finding suggests the contribution of *CsSWEET16* to sugar compartmentation across the vacuole and its function in inducing cold tolerance in tea plants [188]. Thus, manipulating these sugar-transporter genes could regulate sugar levels and ultimately stimulate stress tolerance in transgenic plants.

The potential roles of sucrose-transporter genes *OsSUT1* and *OsSUT2* in photosynthesis and crop-yield improvement was investigated in rice cultivar subjected to elevated CO_2_ concentration [190]. Rice cultivars that respond poorly to high CO_2_ exhibits reduced photosynthetic capacity under elevated CO_2_. The decline in photosynthetic capacity could be strongly associated with the accumulation of soluble sugars. For these cultivars (with poor response to additional CO_2_), increased sink relative to the source strength did not affect photosynthesis. No observed change was detected even with the expression of *OsSUT1* or *OsSUT2*. Contrary to this (poor response cultivars), no such increase in soluble sugars or decline in photosynthesis was observed with “strong” cultivars; instead, elevated CO_2_ increased the expression of the sucrose transporters (*OsSUT1* and *OsSUT2*). It can be inferred from the study that *OsSUT1* and *OsSUT2* expression may be strongly associated with the improvement of photosynthetic capacity in leaf during the grain-filling stage and ultimately increase the yield of rice subjected to high atmospheric CO_2_ [190]. Although researchers have focused more on the influence of environmental cues on the source–sink balance, information on how these stressors affect the activities of sugar transporters and their regulation in mitigating environmental stress is still vague.

## 9. Integrated Approaches to Crop-Yield Improvement

Significant crop-yield-improvement potential has contributed immensely to a rising food demand over the past few decades, consistently keeping pace with rising global demand. The history of crop improvements has been a progressive one, from first- to fourth-generation breeding [191,192,193,194,195]. In the 21st century, the world operates in the fourth generation of breeding. It involves both genome editing and precision breeding, which requires a genetic-engineering technique or gene function to determine crop yields. However, a deep understanding of how this generation of breeding is integrated into the source–sink balance within the plant is yet to be clarified.

Photosynthesis has been relied upon for yield improvement in recent years. Linking specific photosynthetic steps to crop yields seems quite tricky at the source level [192,196]. As such, Wu et al. [197] launched a cross-scale modeling capacity that linked leaf photosynthesis to crop yield in a manner that addresses the conflicting factor in wheat and sorghum raised in water-limiting and water-sufficient conditions. The model was validated using canopy responses and data on crop biomass and yield for wheat and sorghum from various experimental fields. The three major targets included in the model projected for improving crop yields in C3 (wheat), and C4 (sorghum) photosynthesis include: (i) mesophyll conductance for CO_2_, (ii) electron-transport capacity, and (iii) the maximum carboxylation rate of RuBisCO. This model revealed that a boost in each of these components by approximately 20% would individually result in little to no improvement in crop yields. However, a synergetic increase in all three components together boosts the yield of sorghum and wheat to 9.2% and 12.2% under a water-sufficient condition, and with more modest improvement when water is deficient. In recent studies, overexpressing RuBisCO in maize increases the rate of maximal CO_2_ assimilation by 15%, increasing fresh weight. The authors indicated that while no growth disruption was observed under optimal conditions, the overexpressing RuBisCO transgenes could adversely affect yield due to the increased metabolic load [198]. Regulation of stomatal function at the source level has also pointed out some exciting potentials for crop-yield improvement, particularly as stomata offer a promising improvement through water-use efficiency, which is critical to crop-yield improvement. Several other factors improve photosynthetic carbon acquisition and yield at the source level [199,200]. However, whether carbon utilization or limitation in source and sink organs are responsible for crop yields has been a confounding issue over several decades.

Studies have been conducted on the activity of sucrose transport in regulating the source–sink relationship, yet trivial progress has been made in relating this to crop-yield improvements [201]. One of the holistic approaches to improve the yield of crops that use apoplastic phloem-loading would be to uncouple phloem-loading from the sucrose-sensing system, thereby regulating the partitioning of assimilates. There are two rationales behind this approach. The first is the maintenance of constant rates of sucrose removal from the mesophyll by the expression of the sucrose symporter when there is a decline in phloem-loading, and sink demand becomes low. However, the photosynthetic rate still remains high, even on sucrose removal from the mesophyll cells. Secondly, sugar accumulation can trigger leaf senescence [202]. If sugar accumulation induces senescence, the activity of constitutive phloem-loading would reduce the sugar level and subsequently delay senescence. When a reduction in sink demand suppresses photosynthetic rates, and the delay in leaf senescence contributes to a net increase in carboxylation, then maintaining leaf photosynthetic activity depends on the constitutive expression of the symporter. Hence, a net yield increase is realized when this is adopted over a growing season.

Another good approach to yield improvement is overexpressing sucrose transporters in sink cells, which increases the photosynthetic rate and enhances assimilate transport and sink demand. Potato sucrose symporter *StSUT1*, over-expressed in the storage parenchyma cells of developing pea seeds under the control of a vicilin promoter, resulted in enhanced sucrose influx into cotyledons, as well as increased growth rates of the cotyledon [203]. This result demonstrates that engineering a specific promoter for enhanced sucrose transport is quite feasible and can influence the cotyledon growth rate. A related experiment was conducted by Weichert et al. [204], using a Horedin endosperm-specific promoter to overexpress a barley symporter (*Hordeum vulgare*) in grain. Increased grain-protein content and decreased overall yield were observed, although not to a statistically significant level, probably because the experiment was not conducted under a controlled condition. It is worth noting that these authors reported notable changes in gene-expression patterns related to carbon metabolism and amino acid biosynthesis, indicating the abundance of carbon and nitrogen depletion. To this end, it can be inferred that the influence of transgenic manipulation of sucrose transport on multiple metabolic pathways could be a significant means of improving yield.

Further studies have described three major C and N metabolic enzymes, which include *aspartate aminotransferase* (*GmAspAT*), *phosphoenolpyruvate carboxylase* (*ZmPepcase*), and *glutamine synthetase* (*NtGS*), as the best-practice route that alters source–sink interactions [205] when heterologously coexpressed. These aforementioned practice routes improve C and N metabolism, shoot biomass, and seed yield. Even so, the best yield-improvement strategies reported so far have not met the projected need to feed our ever-growing populace by 2050. A significant yield-improvement strategy would be through a series of changes rendered through multigene transformation [206]. An example is a transgenic approach used in alleviating vitamin deficiency affecting up to half of the population in developing countries [206]. Several ongoing projects are currently running this multigene stacking system (employing the Golden Gate toolbox) [18,206].

Two recently developed approaches seem promising regarding our current understanding about the relationship between photosynthesis, plant development, C and N transport, and metabolism. The first is the characterization of transcriptional and metabolic changes to enhance our understanding of the mechanism underlying disrupted source–sink relationships in mutants. This would be a prerequisite to allowing rational engineering of crop yields. The second approach is the modeling strategies and their potential impact on the whole-plant scale, which represents an important testing ground for combined metabolic interventions [8]. In the future, the widespread use of this model seems pragmatic because an overview of the whole-plant physiology will be provided. Other approaches are the adoption of new tools based on hybrid diploid breeding [207,208], speed-breeding techniques [209], and the promising potentials offered by CRISPR [210].

## 10. Conclusions

Plant growth and development are greatly enhanced when the sucrose in the source organ balances with the metabolic needs of the heterotrophic sink. Thus, the strategies involved in improving photosynthetic carbon assimilation for sucrose synthesis and sucrose transport to and its utilization at the sink level become imperative. However, even when these processes involved in sucrose utilization for improved seed (sink) yield are in place, disruptions resulting from environmental cues might emerge. Several questions have arisen as to how photosynthetic activity in both C3 and C4 crops could be further improved or maximized. Such rising issues include: (i) whether evolution has taken over the photosynthetic role in every ecological niche, (ii) whether plants can maximize these ever-increasing CO_2_ concentrations, and (iii) whether breeding programs have influenced the variation between crops for agro-ecosystems and their wild progenitors. All of these questions regarding the potential to fully optimize photosynthesis most likely are still unanswered. In a world of increasing atmospheric carbon-dioxide that is accompanied by environmental deterioration, enhancing sucrose distribution and utilization at the sink is a crucial step in optimizing photosynthesis and maximizing crop yields.

Dynamic regulatory processes mediate the complex interaction of source and sink activity between different organs in the vascular system of the plant. Phloem transport of sugar is closely regulated and highly sensitive to fluctuations in plant environmental cues, resulting in appreciable alterations in the quota of carbon allocated to the sinks. However, there is sparse information on the impact of environmental stress on the phloem transport of photo-assimilates from source to sink organs. Despite the significant roles of sugar transporter in the translocation of sugar within the plants, little is known about how sugar transporter operates under stressed conditions at the molecular level. This is mainly because most studies are mostly tailored toward the physiological responses, rather than the molecular responses, of sugar transporters to these stress factors. Thus, the mechanisms and signals involved in the regulation of source-to-sink activity and their response to the environment should be explored. Knowledge of these will better facilitate further research on the improvement of crop yields, with special focus on sucrose synthesis, transport, and utilization at the sink and underlying environmental cues affecting these processes.

## Data Availability

Not applicable.
